# Psychometric properties and longitudinal measurement invariance of the Functional Assessment of Cancer-Gynecologic Oncology Group-Neurotoxicity subscale in the Chinese colorectal cancer chemotherapy population

**DOI:** 10.1186/s41687-026-01167-1

**Published:** 2026-07-31

**Authors:** Qingchun Geng, Yang Li, Hongwen Ma, Lina Xiong, Jiping Tan, Yanfei Jin

**Affiliations:** 1 School of Nursing, Nanjing Medical University, 101 Longmian Road, Nanjing, Jiangsu 211166 China; 2 Tianjin People’s Hospital, Tianjin, China; 3 The First Affiliated Hospital of Nanchang University, Nanchang, Jiangxi China; 4 The Central Hospital of Enshi Tujia and Miao Autonomous Prefecture, Enshi, Hubei China

**Keywords:** Colorectal cancer, Longitudinal measurement invariance, Measurement invariance, Neuropathy, Oxaliplatin, Psychometric properties, Scale

## Abstract

**Background:**

Chemotherapy-induced peripheral neuropathy is a common and burdensome adverse effect of oxaliplatin-based chemotherapy in patients with colorectal cancer. The Functional Assessment of Cancer Therapy/Gynecologic Oncology Group–Neurotoxicity subscale is used to assess neurotoxicity symptoms, but evidence regarding its psychometric performance and measurement invariance in Chinese patients remains limited. This study aimed to evaluate the psychometric properties and measurement invariance of the Chinese version of the FACT/GOG-Ntx13 in patients with colorectal cancer receiving oxaliplatin-based chemotherapy.

**Methods:**

A longitudinal quantitative survey was conducted among 534 Chinese patients with colorectal cancer. Data were collected one month and three months after the initiation of chemotherapy. Exploratory factor analysis, confirmatory factor analysis, measurement invariance testing, criterion validity analysis, and internal consistency reliability analysis were performed.

**Results:**

Exploratory factor analysis supported a one-factor structure, which was further confirmed by confirmatory factor analysis with acceptable model fit at both time points. The scale demonstrated good criterion-related validity through its positive correlation with the EORTC QLQ-CIPN20 and showed good internal consistency at both assessments. Measurement invariance across gender was supported at configural, metric, and scalar levels. Longitudinally, configural and metric invariance were supported, whereas scalar invariance was not.

**Conclusion:**

The Chinese version of the FACT/GOG-Ntx13 is a valid and reliable instrument for assessing oxaliplatin-induced peripheral neuropathy in patients with colorectal cancer. However, changes in scores over time should be interpreted cautiously because longitudinal scalar invariance was not supported.

**Supplementary Information:**

The online version contains supplementary material available at https://doi.org/10.1186/s41687-026-01167-1.

## Introduction

Chemotherapy-induced peripheral neuropathy (CIPN) is a progressive, persistent, and usually irreversible toxicity of most chemotherapeutic agents, with symptoms including autonomic dysfunction, limb pain, numbness, abnormal pain induced by cold sensation, and nociceptive hypersensitivity [1, 2]. Colorectal cancer (CRC) is the third most commonly diagnosed cancer and the second leading cause of cancer death worldwide [3]. Currently, chemotherapy is an important treatment modality for treating CRC. Oxaliplatin, as a third-generation platinum anticancer drug, is the main chemotherapeutic agent for treating CRC. However, it is also the chemotherapeutic agent that leads to the highest prevalence rate of CIPN, affecting up to 70% of patients [4–6]. Studies have shown that CIPN usually negatively affects patients’ quality of life and physical functioning. In severe cases, it may even interfere with patients’ clinical chemotherapy plans, requiring changes in chemotherapeutic agents and affecting chemotherapy outcomes [7, 8]. Given the widespread use of platinum-based drugs in the treatment of CRC and the severity of CIPN [9, 10], a tool that systematically evaluates CIPN caused by platinum-based drugs is urgently needed for the early identification and effective prevention of CIPN.

Symptoms of CIPN can occur during or after chemotherapy and may worsen with increasing cycles of chemotherapy [11]. Research on CIPN has encompassed a multidimensional range of symptoms from the initial focus on sensory abnormalities such as numbness and tingling sensations to the more generalized symptoms of pain, muscle weakness, autonomic dysfunction, and cold sensitization [12]. Subjective patient reports, professional assessment, and objective neurobiological testing are currently used to assess CIPN. Neurophysiological testing equipment is expensive, complex, and requires expertise and training, making it difficult to scale up in multicenter pilots [13, 14]. Professional assessment to measure changes in clinical symptoms are also difficult because a large amount of score variability in clinician assessments is due to individual differences in assessors [15]. It should be noted that previous research has shown a moderate consistency between patient accounts and physician assessments, and a moderate association between the results of neurobiological testing and patient descriptions of their symptoms [16, 17]. Considering that the impact of symptoms is perceived subjectively, patient-reported outcomes (PROs) are more appropriate for clinical practice than objective neurobiological assessments when tracking adverse events and symptom perception. PROs are also more precise and dependable in capturing a broad spectrum of CIPN symptoms compared to clinician assessments [18].

Symptoms of CIPN progressively worsen with treatment, and there are no specific drugs to prevent CIPN, but several patient-reported outcome measures (PROMs) have been developed to assess CIPN. Existing PROMs have followed a rigorous scientific approach during development to distinguish between different symptoms of CIPN and monitor the progression and severity of neurological dysfunction symptoms. PROs are increasingly recognized as valuable for collecting information on symptoms of CIPN [19–21]. Existing PROMs cover multiple aspects of CIPN and include the European Organization for Research and Treatment of Cancer Quality of Life Questionnaire for Chemotherapy-Induced Peripheral Neuropathy (EORTC QLQ-CIPN20) [22], the Patient Neurotoxicity Questionnaire (PNQ) and physician-based instruments, the National Cancer Institute Common Toxicity Criteria (NCI-CTC) [23]. However, widely used measures such as the PNQ and EORTC QLQ-CIPN20 were not specifically developed to capture oxaliplatin-related manifestations and may therefore be less focused on characteristic symptoms such as cold sensitivity.

The FACT/GOG-Ntx13 was developed especially for the assessment of platinum-induced CIPN by the Gynecologic Oncology Group (GOG) and the Functional Assessment of Chronic Illness Therapy (FACIT) in response to the inadequacy of similar assessment tools in assessing symptoms of platinum-induced CIPN. Owing to the increasing significance of platinum-based chemotherapeutic agents and the rise in cold sensitivity cases associated with them, FACIT developed the 13-item FACT/GOG-Ntx13, specifically for the assessment of oxaliplatin-induced CIPN including sensory, motor, hearing-related, and cold-sensitivity symptoms. The FACT/GOG-Ntx13 has been shown to have high sensitivity in the evaluation of oxaliplatin-induced CIPN [24–26].

However, evidence regarding the psychometric properties of the authorized Chinese 13-item version remains limited, particularly among patients with colorectal cancer receiving oxaliplatin-based chemotherapy. Previous studies have demonstrated that the FACT/GOG-Ntx13 is a reliable and valid instrument; however, further research is needed to examine its psychometric properties in culturally diverse and non-Western populations [24]. Therefore, one aim of the present study was to assess the reliability and validity of the FACT/GOG-Ntx13 in Chinese patients with CRC.

The FACT/GOG-Ntx was developed to assess patient-reported symptoms and concerns associated with chemotherapy-induced neurotoxicity. Its items cover a broad range of clinically relevant manifestations, including sensory disturbances, motor difficulties, hearing-related problems, and cold sensitivity [25, 26]. Although these manifestations represent clinically distinguishable aspects of neurotoxicity, they may also reflect a broader underlying construct of patient-perceived neurotoxicity burden [27]. Existing psychometric studies have not reached a consistent conclusion regarding the factorial structure of the FACT/GOG-Ntx, with different structural solutions reported across studies [28, 29]. This uncertainty highlights the need to further examine whether the instrument primarily reflects a common neurotoxicity construct or captures distinguishable symptom domains across different populations [25]. Accordingly, the present study examined the factorial structure of the FACT/GOG-Ntx13 using exploratory factor analysis (EFA) and confirmatory factor analysis (CFA), and further evaluated whether the identified structure demonstrated stability across groups and over time through measurement invariance (MI) testing.

MI assesses whether a measurement instrument captures the same latent construct across different contexts, such as different groups, occasions, or time points [30]. Establishing longitudinal measurement invariance is as important as assessing reliability and validity, because changes in the factor structure over time may lead to inaccurate inferences in longitudinal studies [31]. Given the rigorous development and iterative refinement of the FACT/GOG-Ntx13, as well as its ability to capture the symptoms and severity of oxaliplatin-induced CIPN, the present study aimed to evaluate the reliability, validity, and temporal stability of the Chinese version of the FACT/GOG-Ntx13. The findings may help support its psychometric validation in non-Western populations and promote its clinical application in China. Prior to the study, the research team obtained authorization from FACIT to use the Chinese version of the FACT/GOG-Ntx13 and to conduct psychometric and longitudinal measurement invariance testing.

## Methods

### Design

A quantitative survey design was used to investigate the psychometric properties and longitudinal measurement invariance of the FACT/GOG-Ntx13. Data were collected using questionnaires at two time points: Time 1, one month after the initiation of chemotherapy, and Time 2, three months after the initiation of chemotherapy.

### Setting and participants

Patients were recruited from the CRC outpatient and inpatient divisions of two tertiary hospitals in Tianjin and Nanchang, China, from June 2024 to November 2024. Inclusion criteria for patients were as follows: (1) had a pathological diagnosis of CRC and were receiving oxaliplatin chemotherapy for the first time; (2) were aged 18 years or older; (3) were able to complete the questionnaire independently; and (4) were voluntary participants. Patients were excluded if they had cognitive or psychiatric impairments, serious heart, liver, or kidney disease, or other severe complications.

### Procedures

The researcher administered the questionnaire face-to-face to participants in the inpatient or outpatient department. Before data collection, research team members received standardized training and used consistent, neutral instructions. Participants were informed of the purpose, content, and importance of the survey and that they could decline to answer any question. If a participant was unable to complete the questionnaire because of visual, literacy or other limitations, a research team member assisted by recording the participant’s responses without suggesting answers. Completed questionnaires were returned immediately and checked on site for completeness. When missing items were identified, participants were invited, but not required, to review them. If a symptom item had been skipped because the symptom was not experienced, the researcher clarified that the lowest response option, “not at all,” indicated the absence of the symptom. Participants then independently decided whether and how to respond. Items that remained unanswered were retained as missing and handled according to the relevant missing-data procedure.

A total of 580 questionnaires were distributed, and 534 valid questionnaires were collected, yielding a valid response rate of 92.1%. The researchers administered the questionnaires at one month (Time 1) and three months (Time 2) after the first chemotherapy treatment.

### Measures

#### Demographics and clinical information

The researchers used a self-designed demographic and clinical information questionnaire to collect participants’ background information, including age, gender, education level, monthly income, place of residence, cancer stage, presence of a stoma, and comorbidities. The questionnaire is provided in Appendix A.

#### The Functional Assessment of Cancer-Gynecologic Oncology Group-Neurotoxicity subscale (FACT/GOG-Ntx13, Chinese version)

The FACT/GOG-Ntx13 is a 13-item instrument designed to assess the severity of oxaliplatin-induced peripheral neuropathy, particularly platinum-related neurotoxicity. The instrument provides an overall neurotoxicity score, although its items cover several symptom manifestations, including sensory, motor/functional, hearing-related, and cold sensitivity symptoms.

The authorized Chinese version was obtained from FACIT. Because an authorized Chinese version was already available, no new translation or back-translation procedure was conducted in the present study. Before the formal survey, a small-scale cultural adaptation and cognitive debriefing process was conducted among patients with colorectal cancer receiving oxaliplatin-based chemotherapy. The results confirmed that the Chinese FACT/GOG-Ntx13 items and response options were clear, understandable, and culturally appropriate for the target population. Additionally, a previous analysis of the T1 data from the same cohort supported the utility of the Chinese FACT/GOG-Ntx13 for the early assessment of CIPN symptoms in patients with colorectal cancer receiving oxaliplatin-based chemotherapy [32]. Its items cover several clinically relevant symptom areas of platinum-induced neurotoxicity, including sensory symptoms, such as numbness, tingling, discomfort, and pain in the hands or feet; motor and functional symptoms, such as general weakness and difficulty fastening buttons; hearing-related symptoms, such as hearing difficulty or tinnitus; and cold sensitivity, including cold-induced discomfort or pain. All items are rated on a 5-point Likert scale ranging from 0 (“not at all”) to 4 (“very much”). Higher total scores indicate more severe oxaliplatin-induced CIPN symptoms [25, 33].

#### European Organization for Research and Treatment of Cancer Quality of Life Chemotherapy-Induced Peripheral Neuropathy Questionnaire (EORTC QLQ-CIPN20)

The EORTC QLQ-CIPN20 is a 20-item instrument comprising three dimensions: sensory, motor, and autonomic domains. A 4-point Likert scale is used to rate each item (1 = “not at all” and 4 = “very much”) [22]. According to the scoring manual, the total score as well as the scores on the three subscales were computed and linearly converted into a 0–100 scale. Higher scores indicate more severe CIPN symptoms.

The Chinese version of the EORTC QLQ-CIPN20 has demonstrated good internal consistency (Cronbach alpha = 0.900 for the overall score, with individual subscales ranging from 0.700 to 0.870) [29].

#### Statistical analyses

Data analysis was performed using IBM SPSS Statistics version 27.0 and IBM SPSS AMOS version 28.0, and multiple-imputation sensitivity analyses were conducted using R version 4.5.1. Descriptive statistics, including frequencies, percentages, means, and standard deviations, were used to describe participants’ demographic and clinical characteristics. Likert-type item scores for the FACT/GOG-Ntx13 and EORTC QLQ-CIPN20 were treated as approximately continuous variables in the statistical analyses [34]. The extent and pattern of missing data were examined before the main analyses. The EFA and Time 1 CFA datasets contained no missing FACT/GOG-Ntx13 item responses. Of the 323 participants in the Time 1 CFA sample, 19 did not complete the Time 2 assessment; therefore, the primary longitudinal analyses were based on 304 complete cases. Attrition analyses compared baseline variables between Time 2 completers and non-completers using independent-samples t tests and chi-square tests, as appropriate. As a sensitivity analysis, 50 multiply imputed datasets were generated under a missing-at-random assumption using multivariate imputation by chained equations with Bayesian linear regression and stochastic posterior draws. The imputation model included all 13 Time 1 and 13 Time 2 FACT/GOG-Ntx13 items. Imputed values were rounded and constrained to the valid 0–4 response range. The Time 2 one-factor CFA and internal consistency analyses were repeated in each dataset, and the mean Time 2 total score was also examined. CFI, TLI, RMSEA, Cronbach’s alpha, and the mean Time 2 total score were summarized descriptively across the 50 imputed datasets and compared with the corresponding complete-case results.

#### Structural validity

For structural validity testing, the Time 1 sample was randomly split into two independent subsamples. The first subsample was used for EFA (*n* = 211), and the second was used for CFA (*n* = 323). The CFA sample was subsequently followed up at Time 2, with 304 participants completing the second assessment. EFA was conducted to examine the underlying latent structure of the FACT/GOG-Ntx13, followed by CFA to evaluate the stability of the factor structure identified by EFA [35]. Principal axis factoring was used to identify the latent construct underlying the 13 neurotoxicity items rather than merely reduce observed variables. An oblique rotation method was specified at the exploratory stage because the symptom areas covered by the FACT/GOG-Ntx13, including sensory, motor/functional, hearing-related, and cold-induced symptoms, are clinically related and may co-occur in patients with oxaliplatin-induced CIPN [36].

Before conducting EFA, the suitability of the data for factor analysis was assessed using the Kaiser–Meyer–Olkin (KMO) measure of sampling adequacy and Bartlett’s test of sphericity. A KMO value of ≥ 0.800 and a significant Bartlett’s test (*p* < 0.001) were considered to indicate that the data were appropriate for factor analysis [37, 38]. The number of factors to retain was not specified a priori but was determined using multiple criteria, including eigenvalues greater than 1.0, visual inspection of the scree plot, and factor-analysis-based parallel analysis. In the parallel analysis, the real-data factor eigenvalues were compared with simulated-data mean eigenvalues generated from random data. Factors were retained when the real-data factor eigenvalues exceeded the corresponding simulated-data mean eigenvalues [39]. The factor structure identified by EFA was subsequently tested using CFA. The following indices were used to evaluate CFA model fit: chi-square (χ²) and its p value, χ²/df, comparative fit index (CFI ≥ 0.900), Tucker–Lewis’s index (TLI ≥ 0.900), and root mean square error of approximation (RMSEA ≤ 0.050). These thresholds were considered to indicate an acceptable model fit [40].

#### Measurement invariance

Measurement invariance of the FACT/GOG-Ntx13 was examined by comparing three increasingly constrained nested models, including configural, metric, and scalar invariance models. All measurement invariance models were estimated using Mplus version 8.3. At Time 1, cross-sectional measurement invariance (CMIs) of the FACT/GOG-Ntx13 was examined using multi-group CFA to determine whether the scale measured the same construct across gender groups, namely male and female patients with CRC receiving oxaliplatin chemotherapy [24]. A series of multi-group CFAs (MGCFA) and longitudinal CFAs (LCFA) were performed to assess the measurement invariance of the FACT/GOG-Ntx13 across gender and over time. We assessed differences in model fit indices between nested models as criteria for measurement invariance. If ΔCFI < 0.010, ΔRMSEA < 0.015, and ΔTLI < 0.010, the scale represented by the model remained constant across clusters [41, 42].

#### Criterion validity

Criterion validity was assessed using Pearson’s correlation coefficient between the FACT/GOG-Ntx13 and the EORTC QLQ-CIPN20.

#### Internal consistency

Internal consistency of the Chinese version of the FACT/GOG-Ntx13 was assessed using Cronbach’s alpha coefficient. A Cronbach’s alpha coefficient > 0.700 was considered to indicate acceptable internal consistency of the total scale [43].

## Results

### Sample characteristics

A total of 534 eligible patients consented to participate in the study. The randomly split EFA and CFA samples included 211 and 323 participants, respectively. Of the 323 participants in the CFA sample, 304 completed the Time 2 assessment, resulting in an attrition rate of 5.88%. Attrition analysis showed no statistically significant differences between completers and non-completers in the examined baseline variables (all *p* > 0.05). The mean age of patients who underwent the EFA was 54.70 (SD = 6.78, range = 35–72), the mean age of patients who underwent the CFA was 52.69 (SD = 8.48, range = 29–80). Demographic and clinical characteristics of the EFA sample, Time 1 CFA sample, and Time 2 CFA sample are shown in Table 1.

**Table 1:** Demographic and clinical characteristics of the samples (*N* = 534)

Variable	EFA (*N* = 211)	Time 1 CFA (*N* = 323)	Time 2 CFA (*N* = 304)
Age (mean ± SD)	54.70 ± 6.78	52.69 ± 8.48	52.68 ± 8.43
Gender			
Male	125(59.24)	150(46.44)	139(45.70)
Female	86(40.76)	173(53.56)	165(54.30)
Education			
Primary or below	66(31.28)	101(31.27)	92(30.26)
Junior high school	75(35.55)	121(37.46)	115(37.83)
Senior high school	52(24.64)	71(21.98)	67(22.04)
College and above	18(8.53)	30(9.29)	30(9.87)
Monthly income (yuan)			
< 3000	73(34.60)	81(25.08)	73(24.01)
3000 ~ 5000	99(46.92)	157(48.61)	150(49.34)
> 5000	39(18.48)	85(26.31)	81(26.64)
Place of residence			
Urban	120(56.87)	192(59.44)	178(58.55)
Rural	91(43.13)	131(40.56)	126(41.45)
Presence of a stoma			
Yes	134(63.51)	148(45.82)	137(45.07)
No	77(36.49)	175(54.18)	167(54.93)
Cancer stage			
Ⅰ	50(23.70)	63(19.50)	56(18.42)
Ⅱ	97(45.97)	132(40.87)	125(41.12)
Ⅲ	45(21.33)	95(29.41)	90(29.61)
Ⅳ	19(9.00)	33(10.22)	33(10.86)
Comorbidity			
Yes	96(45.50)	152(47.06)	141(46.38)
No	115(54.50)	171(52.95)	163(53.62)

Note. M: Mean, SD: Standard deviation

### Missing-data and sensitivity analyses

Time 2 missingness reflected complete wave-level nonresponse rather than sporadic item-level missingness. No significant baseline differences were observed between Time 2 completers and non-completers (all *p* > 0.05). Multiple-imputation sensitivity analyses produced results similar to the complete-case analysis. For the Time 2 one-factor CFA, CFI, TLI, and RMSEA were 0.970, 0.964, and 0.043 in the complete-case analysis and averaged 0.968, 0.962, and 0.044 across the 50 imputed datasets. Cronbach’s alpha was 0.885 versus 0.882, and the mean Time 2 total score was 27.08 versus 27.10. Thus, the principal Time 2 psychometric findings were substantively unchanged.

### Structural validity

#### EFA

**Fig. 1:**
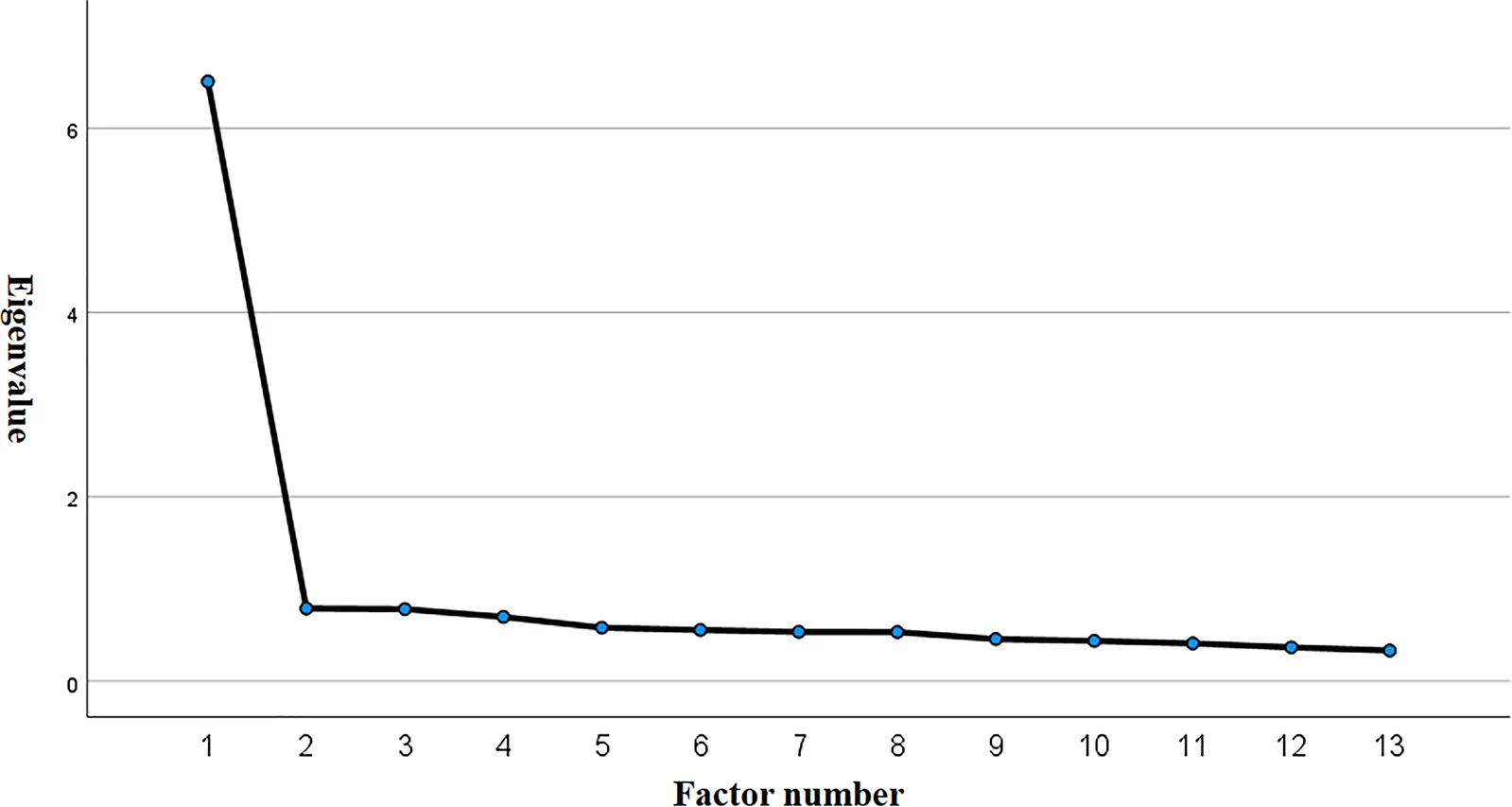
Scree plot of the FACT/GOG-Ntx13

EFA indicated that the data were suitable for factor analysis, with a KMO value of 0.948 and a significant Bartlett’s test of sphericity (χ² = 1214.091, df = 78, *p* < 0.001). Using principal axis factoring, a one-factor solution with an eigenvalue greater than 1.0 was retained. The first four observed eigenvalues were 6.506, 0.791, 0.784, and 0.699, respectively. Both the scree plot and FA-based parallel analysis supported the one-factor solution, as only the first factor was suggested for retention in the parallel analysis. The extracted factor explained 45.913% of the total variance, and all 13 items had factor loadings above 0.600, ranging from 0.615 to 0.725. The scree plot of the FACT/GOG-Ntx13 is presented in Fig. 1, and the factor loading matrix is shown in Table 2.

**Table 2:** Factor loadings from principal axis factoring (*N* = 211)

Item	Factor loading
1	0.643
2	0.691
3	0.678
4	0.665
5	0.700
6	0.693
7	0.702
8	0.698
9	0.644
10	0.725
11	0.703
12	0.615
13	0.643
Variance explained (%)	45.913

#### CFA

**Fig. 2:**
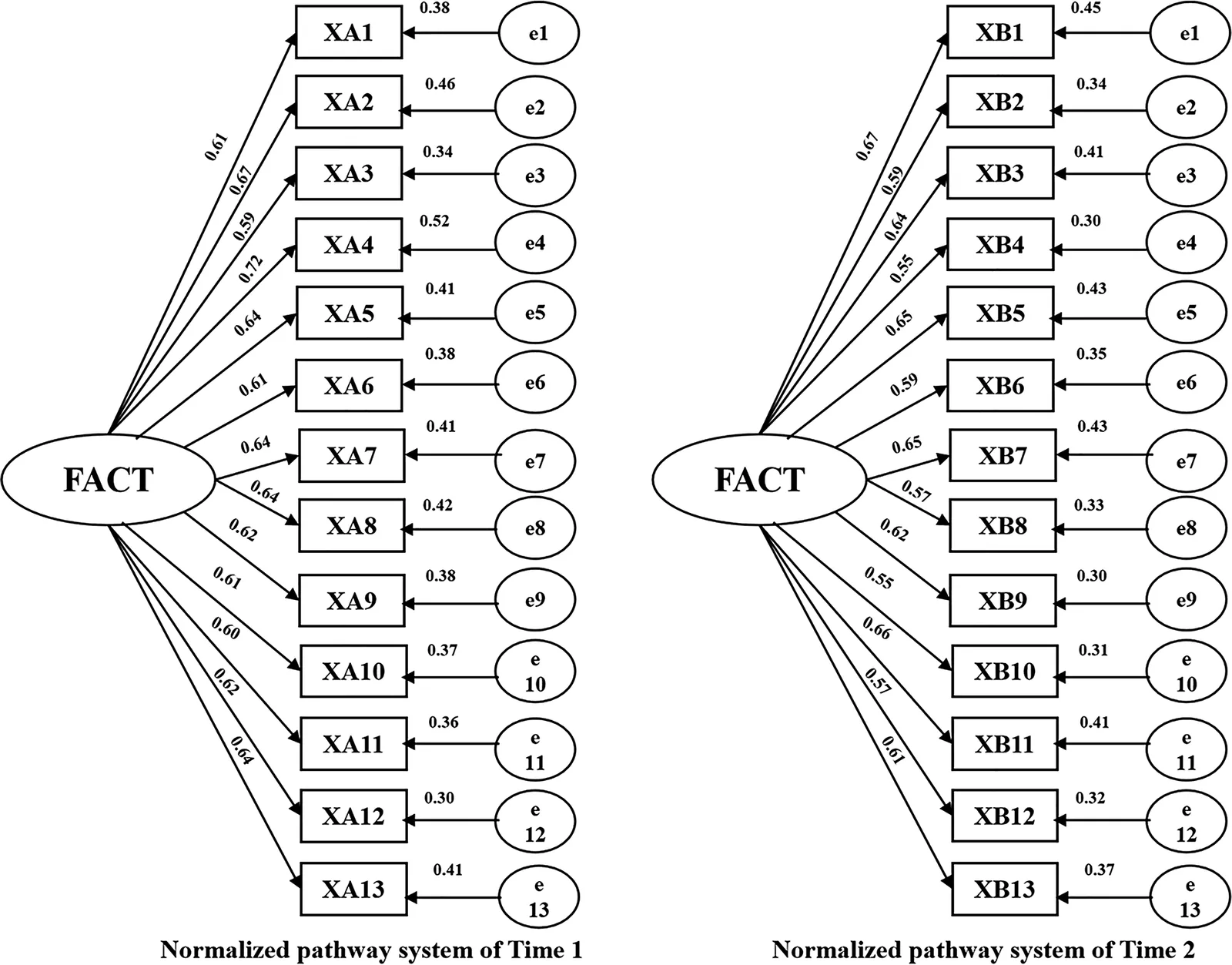
Standardized one-factor model of the Chinese version of the FACT/GOG-Ntx13 at Time 1 and Time 2. Note: X1-X13 are items in FACT/GOG-Ntx13

CFA supported a one-factor model of the FACT/GOG-Ntx13 at both Time 1 and Time 2. All observed items were retained as indicators of the latent factor. The one-factor CFA model demonstrated good fit: Time 1, χ²/df = 1.532, *p* < 0.001, TLI = 0.999, CFI = 0.976, RMSEA = 0.041; Time 2, χ²/df = 1.552, *p* < 0.001, TLI = 0.964, CFI = 0.970, RMSEA = 0.043. The factor structure and standardized path coefficients are presented in Fig. 2.

### Criterion validity

Criterion validity was assessed using Pearson’s correlation between FACT/GOG-Ntx13 and EORTC QLQ-CIPN20. Due to the lack of evidence for longitudinal measurement invariance and questions regarding the structure of the EORTC QLQ-CIPN20 [16, 44], correlations were calculated only at Time 1. The FACT/GOG-Ntx13 demonstrated a significant positive correlation with the EORTC QLQ-CIPN20 (*r* = 0.787, *p* < 0.001), indicating good criterion-related validity.

### Internal consistency

Internal consistency of the FACT/GOG-Ntx13 was assessed using Cronbach’s alpha at both Time 1 and Time 2. The alpha coefficients were 0.896 and 0.885, respectively, indicating good internal consistency.

### Measurement invariance

At Time 1, measurement invariance across gender was supported. The configural model demonstrated acceptable fit across male and female participants (χ² = 185.807, *p* < 0.001, RMSEA = 0.052, CFI = 0.962). The metric and scalar invariance models also met the recommended criteria, indicating that the factor structure, factor loadings, and item intercepts were comparable across gender groups.

Longitudinal measurement invariance was then assessed across Time 1 and Time 2. The configural and metric invariance models were supported, indicating that the one-factor structure and factor loadings were stable over time. However, the scalar invariance model was not supported, as the changes in model fit exceeded the recommended cut-off values (ΔCFI = 0.057, ΔTLI = 0.055, and ΔRMSEA = 0.027). These findings suggest that item intercepts were not fully invariant across Time 1 and Time 2. Fit statistics for each model are presented in Table 3.

**Table 3:** Measurement invariance of the one-factor FACT/GOG-Ntx13 model

Hypothesis	χ² (df)	CFI	ΔCFI	TLI	ΔTLI	RMSEA	ΔRMSEA
**Gender (Male vs. Female)**							
Configural Model	185.807(130)	0.962		0.955		0.052	
Metric Model	198.572(142)	0.962	0	0.958	0.003	0.050	0.002
Scalar Model	215.784(154)	0.958	0.004	0.958	0	0.050	0
**Time (T1 vs. T2)**							
Configural Model	197.501(130)	0.975		0.970		0.041	
Metric Model	216.658(142)	0.972	0.003	0.969	0.001	0.041	0
Scalar Model	379.321(154)	0.915	0.057	0.914	0.055	0.068	0.027

Note. χ² = chi-square; df = degrees of freedom; CFI = comparative fit index; TLI = Tucker–Lewis index; RMSEA = root mean square error of approximation; Δ = change in model fit index compared with the preceding model; T1 = 1 month after initial chemotherapy; T2 = 3 months after initial chemotherapy

## Discussion

This study provides evidence regarding the structural validity and measurement invariance of the Chinese FACT/GOG-Ntx13 in patients receiving oxaliplatin-based chemotherapy. A one-factor structure was supported, and the scale showed good internal consistency and a strong association with the EORTC QLQ-CIPN20. Although measurement equivalence was established across gender, longitudinal scalar invariance was not supported, indicating that changes in observed scores may not reflect only changes in the underlying neurotoxicity construct.

EFA supported a one-factor structure for the Chinese FACT/GOG-Ntx13, which was further confirmed by CFA at both Time 1 and Time 2. Although the scale covers clinically distinguishable sensory, motor or functional, hearing-related, and cold-sensitivity symptoms, the present findings suggest that these manifestations share a common underlying component reflecting patients’ overall perception of chemotherapy-related neurotoxicity burden [27]. This result extends previous evidence that the proposed multidimensional structure of the FACT/GOG-Ntx has not been consistently supported, as neither the four-factor model nor an alternative three-factor solution demonstrated stable support across assessment points [29, 45]. Therefore, the FACT/GOG-Ntx13 total score may be interpreted as an overall indicator of patient-reported neurotoxicity severity rather than as separate symptom subscales. Nevertheless, the identification of a general neurotoxicity factor does not imply that the scale fully captures all clinically relevant aspects of chemotherapy-induced neurotoxicity [16]. Further studies incorporating related symptom measures and objective neurological assessments are needed to clarify the construct boundaries and discriminant validity of the FACT/GOG-Ntx13.

Previous studies of the FACT/GOG-Ntx13 have mainly focused on reliability, validity, and factor structure, whereas evidence regarding measurement invariance has been limited [29]. Recent oncology psychometric studies have increasingly emphasized the importance of testing measurement invariance across patient groups and over time to support valid comparisons [46, 47]. Previous studies have suggested that gender may be associated with variations in the occurrence and severity of chemotherapy-induced peripheral neuropathy; however, the underlying mechanisms remain incompletely understood [48]. The present study extends previous psychometric evidence by providing evidence that configural, metric, and scalar invariance were supported across gender. This indicates that the Chinese FACT/GOG-Ntx13 assesses the same underlying construct with comparable factor loadings and item intercepts across male and female patients, supporting meaningful gender-based comparisons.

Longitudinal analyses demonstrated configural and metric invariance between Time 1 and Time 2, indicating that the underlying neurotoxicity construct and the relationships between items and the latent factor remained stable during chemotherapy. These findings extend previous longitudinal validation studies of the FACT/GOG-Ntx, which demonstrated satisfactory reliability, validity, and responsiveness over time, by providing additional evidence regarding longitudinal measurement invariance [29] However, scalar invariance was not supported, indicating that item intercepts may have varied over time. Because scalar invariance is important for interpreting mean differences across groups or time points, changes in FACT/GOG-Ntx13 scores over time should be interpreted with caution unless further evidence of partial or full scalar invariance is established [49].

The strong correlation between the FACT/GOG-Ntx13 and the EORTC QLQ-CIPN20 supports the criterion-related validity of the Chinese version of the FACT/GOG-Ntx13. Although both instruments assess CIPN, the FACT/GOG-Ntx13 was specifically developed for platinum-associated neurotoxicity and includes cold sensitivity, a characteristic symptom of oxaliplatin-induced neurotoxicity [50]. Previous studies have also demonstrated its sensitivity and reliability in assessing chemotherapy-related neurotoxicity [24, 51]. Therefore, the FACT/GOG-Ntx13 may serve as a useful patient-reported measure for assessing neurotoxicity burden in patients receiving oxaliplatin-based chemotherapy. The high internal consistency observed at both time points further supports the reliability of the instrument across assessment occasions.

### **Limitations and future directions**

Several limitations should be acknowledged. First, participants were recruited from two tertiary hospitals in Tianjin and Nanchang, China, which may limit the generalizability of the findings. The inclusion of both inpatient and outpatient participants may have enhanced sample diversity; however, future multicenter studies are needed for further validation. Second, criterion validity was assessed only at Time 1 because longitudinal validation data for the EORTC QLQ-CIPN20 were unavailable, limiting the evaluation of longitudinal validity. Future studies should include additional validated CIPN measures to further examine longitudinal validity. Third, this study focused on oxaliplatin-induced peripheral neuropathy in patients with colorectal cancer, and the findings may not be generalizable to CIPN caused by other chemotherapy regimens. Future research should examine the applicability of the FACT/GOG-Ntx13 across different populations and neurotoxic agents. Finally, potential demographic and treatment-related factors associated with CIPN severity were not explored. Future studies should investigate these factors to better understand variations in CIPN symptoms.

## Conclusion

The Chinese FACT/GOG-Ntx13 demonstrated a well-supported one-factor structure, good internal consistency, and full measurement invariance across gender in patients with colorectal cancer receiving oxaliplatin-based chemotherapy. These findings support its use for assessing overall neurotoxicity burden and for meaningful comparisons between male and female patients. Although longitudinal configural and metric invariance were supported, the absence of scalar invariance indicates that changes in observed scores over time should be interpreted cautiously, as they may not solely reflect changes in underlying neurotoxicity. 

## Supplementary Information

Below is the link to the electronic supplementary material.

Supplementary Material 1

Supplementary Material 2

## Data Availability

The data that support the findings of this study are available from the corresponding author upon reasonable request.
