# Supplementary material for: Psychometric properties and longitudinal measurement invariance of the Functional Assessment of Cancer-Gynecologic Oncology Group-Neurotoxicity subscale in the Chinese colorectal cancer chemotherapy population
Source: J Patient Rep Outcomes. 2026 Jul 31;10:198. doi: 10.1186/s41687-026-01167-1 (PMC13724603; doi:10.1186/s41687-026-01167-1)
Supplement: Supplementary file 1 [file 41687_2026_1167_MOESM1_ESM.docx]

**Table for FACT/GOG-Ntx13-item in Chinese and English**

| **Item** | **Original English version** | **Target Chinese version** |
| --- | --- | --- |
| **1** | I have numbness or tingling in my hands | 我的手有麻木或刺痛的感觉 |
| **2** | I have numbness or tingling in my feet | 我的脚有麻木或刺痛的感觉 |
| **3** | I feel discomfort in my hands | 我的手有不舒服的感觉 |
| **4** | I feel discomfort in my feet | 我的脚有不舒服的感觉 |
| **5** | I have joint pain or muscle cramps | 我关节痛或肌肉痉挛 |
| **6** | I feel weak all over | 我觉得全身虚弱无力 |
| **7** | I have trouble hearing | 我的听力有问题 |
| **8** | I get a ringing or buzzing in my ears | 我耳朵里有嗡嗡声或耳鸣 |
| **9** | I have trouble buttoning buttons | 我扣扣子有困难 |
| **10** | I have trouble feeling the shape of small objects when they are in my hand | 我拿着小东西时，不容易摸出它的形状 |
| **11** | I have trouble walking | 我走路有困难 |
| **12** | I have pain in my hands or feet when I am exposed to cold temperatures | 当我暴露在寒冷的气温下时，我的手或脚会感到疼痛 |
| **13** | I have difficulty breathing when I am exposed to cold temperatures | 当我暴露在寒冷的气温下时，我会呼吸困难 |
