# Supplementary material for: Psychometric properties and longitudinal measurement invariance of the Functional Assessment of Cancer-Gynecologic Oncology Group-Neurotoxicity subscale in the Chinese colorectal cancer chemotherapy population
Source: J Patient Rep Outcomes. 2026 Jul 31;10:198. doi: 10.1186/s41687-026-01167-1 (PMC13724603; doi:10.1186/s41687-026-01167-1)
Supplement: Supplementary file 2 [file 41687_2026_1167_MOESM2_ESM.docx]

**Appendix A**

**Demographic and Clinical Information Questionnaire**

Instructions: Please select the appropriate option or fill in the blank according to the participant’s information.

| Item | Response options |
| --- | --- |
| Gender | □ Male □ Female |
| Age | ______ years |
| Education level | □ Primary or below  □ Junior high school  □ Senior high school  □ College or above |
| Monthly income (yuan) | □ < 3000 □ 3000–5000 □ > 5000 |
| Place of residence | □ Urban □ Rural |
| Cancer stage | □ Stage I □ Stage II □ Stage III □ Stage IV |
| Presence of a stoma | □ Yes □ No |
| Comorbidities | □ Yes □ No |

**Note.** This questionnaire was developed by the research team to collect participants’ demographic and clinical information.
